# Discrete classification technique applied to TV advertisements liking recognition system based on low-cost EEG headsets

**DOI:** 10.1186/s12938-016-0181-2

**Published:** 2016-07-15

**Authors:** Luis M. Soria Morillo, Juan A. Alvarez-Garcia, Luis Gonzalez-Abril, Juan A. Ortega Ramírez

**Affiliations:** 1 Computer Languages and Systems Dept, University of Seville, Avda. Reina Mercedes s/n, 41012 Seville, Spain; 2 Applied Economics I Dept, University of Seville, Avda. Ramon y Cajal, 1, 41018 Seville, Spain

**Keywords:** Neuromarketing, Electroencephalography, Advertising, EEG, Brain-computer interaction

## Abstract

**Background:**

In this paper a new approach is applied to the area of marketing research. The aim of this paper is to recognize how brain activity responds during the visualization of short video advertisements using discrete classification techniques. By means of low cost electroencephalography devices (EEG), the activation level of some brain regions have been studied while the ads are shown to users. We may wonder about how useful is the use of neuroscience knowledge in marketing, or what could provide neuroscience to marketing sector, or why this approach can improve the accuracy and the final user acceptance compared to other works.

**Methods:**

By using discrete techniques over EEG frequency bands of a generated dataset, C4.5, ANN and the new recognition system based on Ameva, a discretization algorithm, is applied to obtain the score given by subjects to each TV ad.

**Results:**

The proposed technique allows to reach more than 75 % of accuracy, which is an excellent result taking into account the typology of EEG sensors used in this work. Furthermore, the time consumption of the algorithm proposed is reduced up to 30 % compared to other techniques presented in this paper.

**Conclusions:**

This bring about a battery lifetime improvement on the devices where the algorithm is running, extending the experience in the ubiquitous context where the new approach has been tested.

## Background

Electroencephalography has been used over the last few years for the study of neural oscillat1ions produced by neurological activity. In several studies related with neurology science, the main goal is to determine the effects of some kinds of feelings and sensations in the brain cortex. Thus, cause-effect relationships to some external events have been established, evidenced by the neural activity in the brain cortex. The combination of medicine and learning systems developed in computer science have made possible to perform unbelievable aims some years ago [1–5]. Since the increasing use of electroencephalographs in brain-computer interface applications and the capability of isolate the correlation between electrical potentials on the scalp and different feelings, several works have been done in this field [6–8]. Most of them use fMRI (functional magnetic resonance imaging) or expensive EEG devices to obtain and process the neural oscillations, so called brain waves. This has led to a new age in the research on human-computer interactions. This field is characterized by the absence of physical movements of the user to execute a set of action. Instead, the interaction is based on thoughts and brain states [9–11]. In an effort to isolate the actions, for instance, Some works [12–14] have found associations to determine whether the music heard is pleasant or unpleasant according to the subjects’ interest, or if a short TV advertisement get or not the user attention [15–17]. This application is being exploited nowadays by the advertising industry. Based on these works, this paper is focused on the relationship between the visualization of short TV advertisements and the effects they cause on the mental state of the user, represented by the brain activity observed throughout EEG devices. Specifically, this study aims to provide a solution to a recurring problem in neuromarketing: to determine marketing preferences without physical user interaction and in a completely objective way. This aim will answer to questions as how is the brain cortex behaviour when a user watch an ad that he/she likes? What happens when dislikes? Is there some electric effect when the user is watching an advertisement that fits with his or her interests? [18–20]. And finally, even more important than previous questions, is there anyway to integrate nowadays this kind of systems in subjects’ daily life? So far, most studies in the field of neuromarketing have been based on the use of electroencephalographs or galvanic skin resistance sensor to determine the impact of ads in the neural activity manifested in the brain waves [21, 22]. Furthermore, some of them use this technology to classify input data (digitalized value obtained from EEG electrodes) into binomial classes, such as [I like] or [I hate]. In this paper, the development of a learning system based on Ameva discretization algorithm will be presented. This approach is compared with other techniques present on the state of the art. Concretely, neural networks [23, 24] and decision trees (C4.5) [25] are used to perform this comparison. All three algorithms are applied in order to discover patterns to classify the level of interest of subjects in a set of video advertisements. The proposal is looking for improving the speed of the classification phase, making possible to develop real-time systems for this purpose. Furthermore, this work faces up the EEG data classification problem from a completely new perspective. It’s one of the first time that discrete approaches are used in this division of knowledge and even more, using low cost EEG sensors. The importance of using low cost EEG sensors gives rise to this solution. The aim of the system described in this paper is to integrate the EEGs usage in the subjects’ daily life. However, one of the main barriers to the presence of these devices in society is, among other, the high-cost of necessary hardware and the size of the professional solutions. For this reason, most studies done in this field have been performed in clinical environments or in laboratories. Instead, this approach brings the possibility of collect information from anywhere and at anytime, even if the subject is watching TV or working with computer. Concretely, in the area of human likes’ research, this is really important in order to avoid external disturbance factors in their feelings. By hence, in the scenario proposed in this paper, where short TV commercials interest for users are under study, this solution is very convenient and the benefits of this approach are unmistakable. There are also a lot of applications based on brain-computer interfaces working in home environments. Home automation and applications may be fostered by the massive use of this technology [26–28]. Nowadays, thanks largely to frameworks such as OSGi [29], providers can connect context of this typology in an easy way to larger software architectures. The first psychological study that use EEG, dates back to 1979 [30]. This one and the subsequent studies, validate that electrical patterns were lateralized in the frontal region of the brain. Generally, the measure of alpha-band waves (8–13 Hz) in the left frontal lobe indicates positive emotions. Furthermore, high activity in parietal area during the observation of the TV commercials with an affective content was also noted in other works [31, 32]. Based on previous works [33], parietal and superior prefrontal regions are associated with the maintenance of highly processed representation of complex stimuli, such as TV advertisements. The remainder of this paper is organized as follows. “Materials and dataset” section shows experiment conditions and the hardware configuration to create the classification dataset, as well as other applications of brain-computer interfaces. In “Materials and dataset” section is also presented the feature extraction model, the dataset obtained from EEG sensors, and all cognitive evaluations that can be recognized by the presented system. “Methods” section presents two methods for EEG data classification used in the state of the art, and a new approach based on the Ameva [34] discretization algorithm. In “Description of the Ameva system” section, the classification process and an improvement of the discretion algorithm used are presented. “Results” section discusses the advantages of the proposed algorithm and the results of the comparison among all three methods presented in “Methods” section. Finally, “Conclusions” section states some challenges and future works related to the EEG recognition system presented.

## Materials and dataset

### Materials

**Fig. 1:**
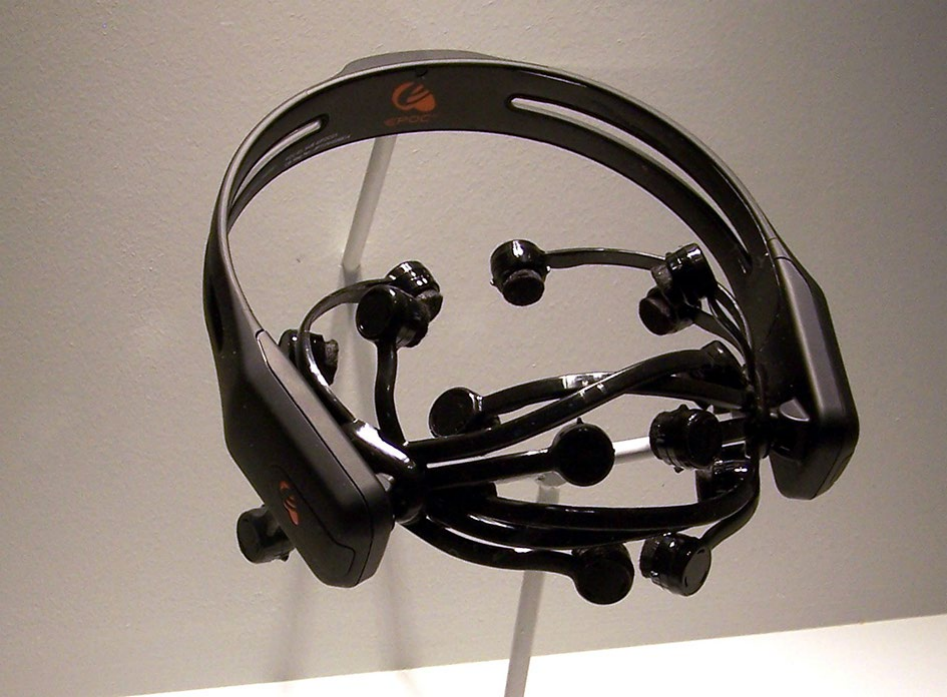
Emotiv EPOC device

In this study a low-resolution sensor with an affordable price is used to predict the rating given to a set of TV video advertisements by different subjects [2]. This rating is associated to the liking level of each video for each user. In this study, the Emotiv EPOC (Emotiv Systems, Inc., San Francisco, CA) electroencephalograph will be used to obtain the brain waves during the visualization (1). However, there are several startups that have products similar to Emotiv EPOC under development with almost the same features, and some of them even smaller. Many of these products is having a similar appearance to a wireless headphones [35, 36], which makes that the user can wear them in a constantly. This step towards wearable electroencephalographs will require the use of simple devices and efficient algorithms to determine several aspects and relationships between neural activity and external events, feelings and emotions. If this objective is reached, people will be able to use this sensor in the same way they use other wearables. The Emotiv EPOC headset and its Software Development Kit for research include 14 channels (plus CMS/DRL references, P3/P4 locations) each based on saline sensors. This headset has not the ability to cope with all the BCI paradigms with the same success without modifying the hardware. For instance, the motor imagery paradigm, which requires central electrodes, should provide bad performance. As shown in Fig. 1, the headset is completely wireless and has a large Lithium-based battery autonomy of 12 h. The sampling rate can reach 128 Hz. Additionally, gyroscope generates positional information for cursor and camera controls among other applications. In this work, all the standard available electrodes of the Emotiv EPOC headset were used. Electrode impedance was decreased by using saline liquid until the level required in order to improve the skin contact and system conductivity. Furthermore, during the experiments the contact area of each sensor was moistened by a conductive gel.

### Dataset

**Fig. 2:**
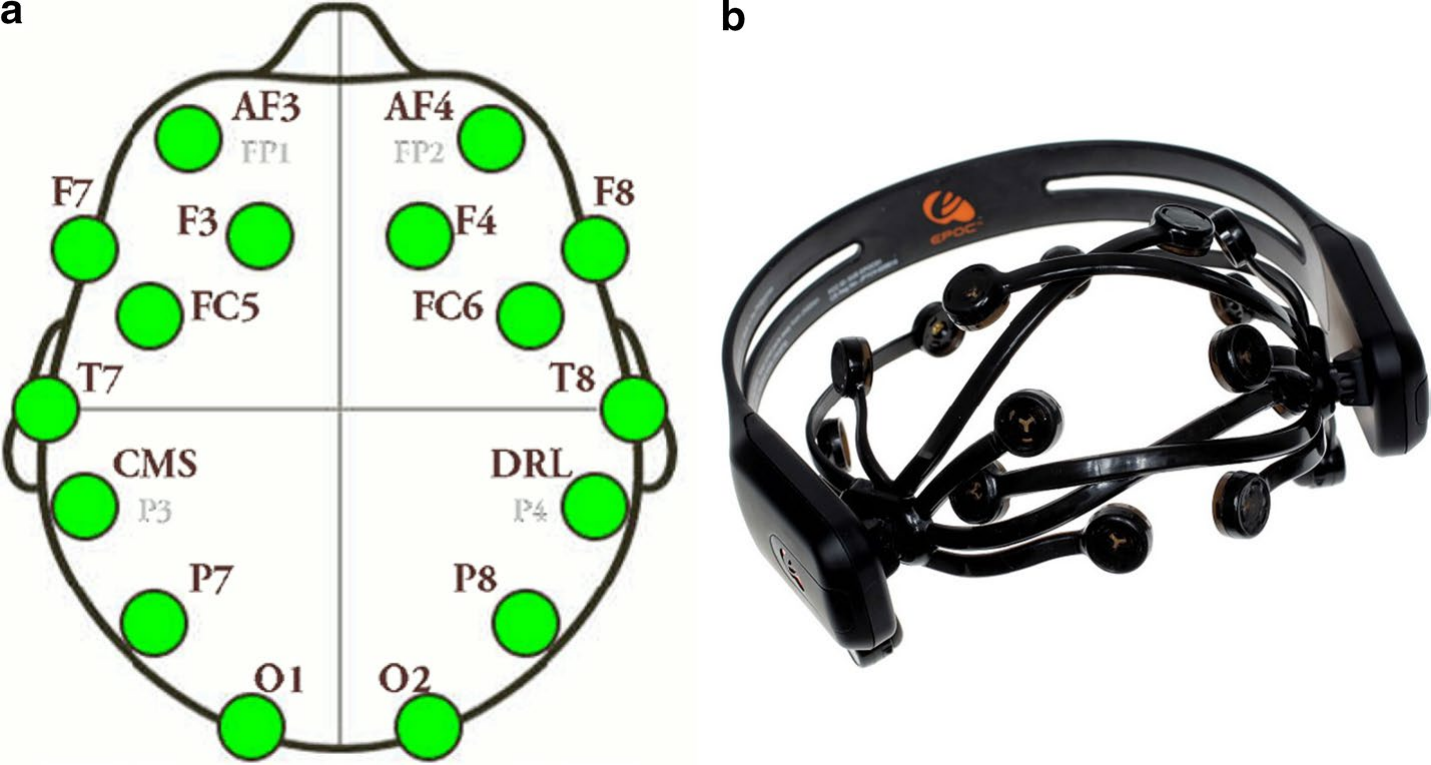
Emotiv EPOC characteristics and sensors configuration. **a** 10–20 International system configuration, **b** headset and sensors

The algorithms introduced in this paper for the human-like recognition system have been performed and evaluated by means of a custom dataset created during the experiments. This dataset has been built by showing a set of 14 short TV advertisements to 10 subjects of different age and gender. The experiment was carried out in a laboratory with temperature and illumination under control. EEG signals were recorded using an Emotiv EPOC headset system through 14 passive moist electrodes with a sampling frequency of 128 Hz (2048 Hz internal). These electrodes was arranged according to the international 10–20 system, including electrodes at AF3, F7, F3, FC5, T7, P7, O1, O2, P8, T8, FC6, F4, F8, and AF4 positions. Figure 2 shows electrode configuration used by Emotiv EPOC device. All 10 subjects were asked for the like degree of each TV advertisement, rating them between 1 and 5 (where 1 represents “I didn’t like at all”, and 5 stands for “I’d like a lot”). By other side, subjects were requested that they wrote down the remembered TV ads once the session completed. Short TV advertisements were carefully selected among a set of 150 videos. TV advertisements were chosen based on the impact of each one in the subjects responsible for the advertisements selection. This process was made before the training and evaluation process by different users. Before each session beginning, a 15 s baseline was recorded in order to correct the EEG signal from stimulus-unrelated variations in power over time.

With the aim of reduce the noise in the EEG signal and identify the correlation between subjective ratings and the electroencephalographic signal obtained, a high-pass filter with a 2 Hz cutoff frequency was applied. Furthermore a low-pass filtering process with a 47 Hz cutoff frequency was performed from the RAW signal by means of a IIR Butterwoth filter. This process is executed offline and the whole data sequence is available prior to filtering. Following on from this premise, IIR nonlinear phase behaviour does’t represent a problem in our system. In order to avoid this problem in real-time and continuous execution of this approach, a system based on sliding windows will be presented afterward.

Finally, some epochs were contaminated with activity that does not come from brain processes. This is the case of the eye-blinking artifacts and muscle activation provoked by facial movements. These and other clearly identified artifacts were removed using RunICA algorithm implemented in EEGLAB toolbox [37].

All 14 short videos were between 20 and 60s long (M = 30.8 s, SD = 14.2 s). Although psychologists recommend videos from one to ten minutes long for single emotion recognition studies [38], in this study video clips were kept as short as possible to avoid multiple emotions or habituation to the stimuli. Furthermore, this is the most common TV ad length nowadays. Accordingly, this choice represents a good approach to any real environment.

The distribution of subjects in the experiment is shown in Table 1.

**Table 1:** Subject profile on the data recovery process

Subject	Age (years)	Gender	Concentration level
1	28	Man	High
2	31	Woman	Medium
3	27	Woman	Medium
4	26	Man	High
5	54	Man	Low
6	59	Woman	Medium
7	60	Man	High
8	63	Woman	Low
9	42	Man	High
10	46	Woman	Medium

The concentration level refers to the attitude presented by the subject before and during the test process. This attribute take three different values: high (high interest in performing the test and low degree of distractions), medium (high interest in performing the test and noticeable impact of external distractions) and low (low interest during the test process and high impact of outer disturbances). Subjects with different values in this attribute have been selected to check the robustness of the algorithm. The dataset was generated automatically by an Android app developed for this purpose. The application stores the videos that will be played. Once the videos have been played, application inquiries to indicate which videos the user reminds (this can be understood as an indicator of the impact of the video on the user). Then, description of each video is shown to the users, who will rate each ad. The result is an XML file that contains the subject’s profile, temporal information about the beginning and the end of each video, the set of remembered advertisements right after completion of the test, rating for each video and, finally, information about each TV advertisement.
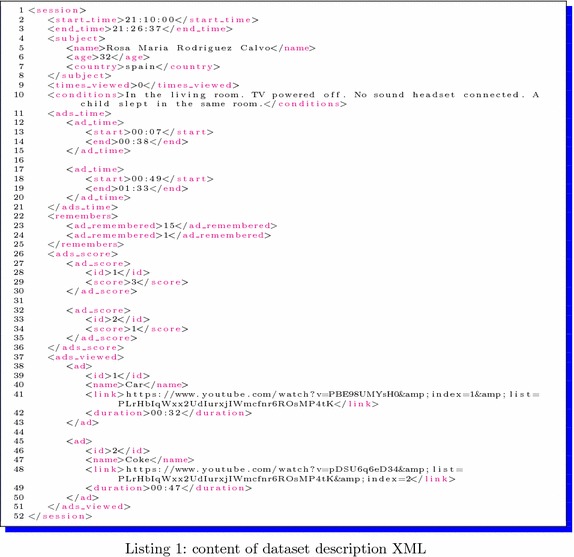


**Fig. 3:**
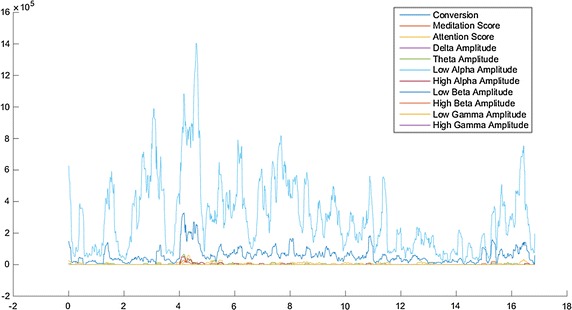
EEG data from Emotiv EPOC

All the information about the session visualization process is represented by means of this XML file. This data will be important when the learning process is conducted. On the other hand, while the user proceeds watching the content, neural activity associated is collected using the Emotiv EPOC headset. All electric levels delivered by the EEG are stored in the mobile phone in order to obtain new features from them and to apply the rating estimation algorithm. Signals recovered are meditation, attention, delta amplitude, theta amplitude, low alpha amplitude, high alpha amplitude, low beta amplitude, high beta amplitude, low gamma amplitude and high gamma amplitude. This information can be seen in Fig. 3.

**Fig. 4:**
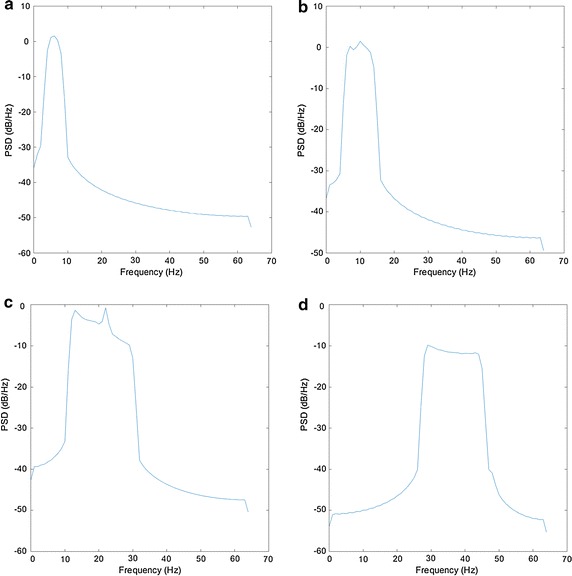
Results of the *PSD* extraction processing for each band. In the abscissa *axis* is shown de power of the signal per Hz in dB/Hz. In the ordinate axis, frequencies are shown in Hz. **a** PSD for alpha channel (3–7 Hz), **b** PSD for beta channel (8–13 Hz), **c** PSD for gamma channel (14–29 Hz) **d** PSD for theta channel (30–47 Hz)

Starting from the RAW data delivered by the EEG sensor, a filtering process was performed. Each filter will provide brain wave profiles linked to the most important frequency bands of brain activity. These bands are theta (3–7 Hz), alpha (8–13 Hz), beta (14–29 Hz), and gamma (30–47 Hz). From this database is possible to obtain a total of 1064 statistical sampling parameters (19 for each band and channel) using values associated to previous frequencies. These parameters will be considered hereafter as inputs of the classification system. Thanks to statistical values obtained from the original signal, discrete relationships can be established between instances with no temporary data processing needed. As will be discussed later, by one side, this allows to reduce the complexity of the learning and classification process and by other side, results will be easy to understand. In addition to previous statistical parameters, 4 more inputs per EEG channel were included. These inputs corresponds to the logarithm of the power spectral density values (*PSD*) of each frequency band introduced previously [39, 40]. *PSD* was computed using the Welch’s power spectral density estimate. The result of this processing is shown in Fig. 4. From this signal, the frequency power of trials and baselines between 3 and 47 Hz was extracted with windows of 128 samples and an overlapping of 64 samples. The baseline power obtained from the *PSD* was removed from the signal obtained during the experiment for each subject. Finally, the difference between the spectral power of all 7 symmetrical pairs on the right and left hemisphere was extracted to measure the possible asymmetry in the brain activity due to the valence of an emotional stimuli. From both *PSD* and symmetrical pairs, 133 new inputs were added to the system (19 for each symmetrical pair and *PSD* sequence). Therefore, this makes a total amount of 1242 statistics per window.

Finally, in order to increase the general accuracy of the system, the difference between the spectral power of all the 7 symmetrical pairs on the right and left hemisphere was extracted [41]. This new data will be used to improve the measurements of pleasantness score from EEG data due to the possible asymmetry in the brain activities due to the valence of an emotional stimuli. The asymmetry structures were obtained from frequency bands described previously. Finally, based on some studies where have been discussed the relationships between symmetrical channels [2], it has been calculated the *Z-index*. This index give an objective value about the correlations between different channels. This value is calculated as follow:

1 $$\begin{aligned} Z = \dfrac{\overline{X} - \mu }{\sigma / \sqrt{N}} \end{aligned}$$

where X refers to the distribution of *PSD* values (of cardinality N) obtained during the observation of each video and $\overline{X}$ is the mean of these values. $\mu$ denotes the mean value of *PSD* activity over the whole session and $\sigma$ its standard deviation. Once this value is calculated for each symmetric pair channel, Person correlation coefficient is obtained for pleasant and unpleasant videos independently. The result of this correlation is added too as input value for the system. In order to avoid interferences generated by external stimuli and other kind of artifacts, 10 s (4 s overlapped) time windows are defined in this process. Therefore, in a common 30 s TV ad, 5 time windows will be produced.

## Methods

**Fig. 5:**
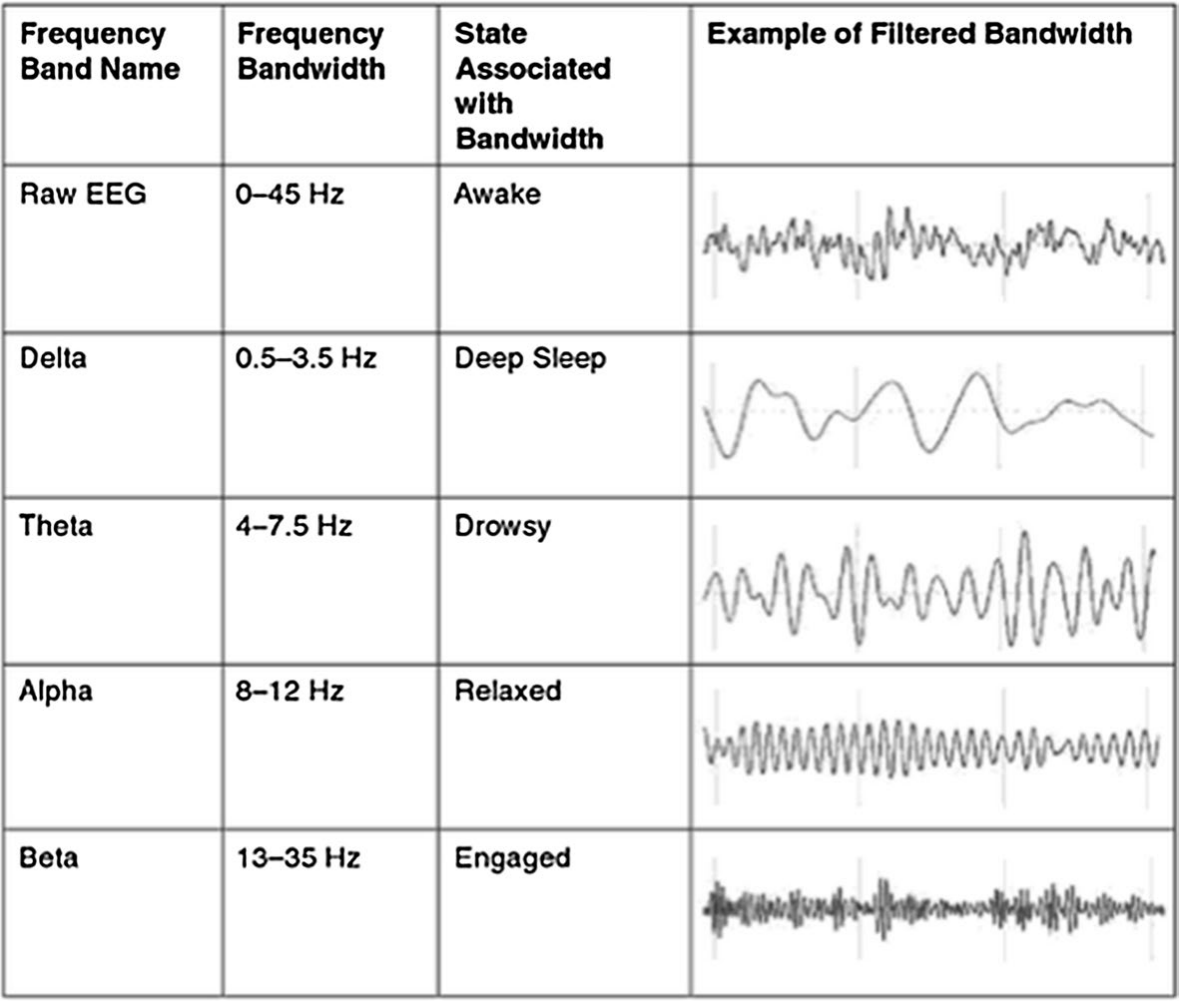
Common bands in EEG signal and example of filtered wave

Commonly, standard EEG bands are associated to different brain states. Figure 5 shows most used bands in the brain study and the state associated to each one. An example of the filtered RAW signal is shown in order to compare this behaviour with the results presented below during the algorithm execution.

Previous signals allow the extraction of a set of features to classify the dataset of TV advertisements in 5 categories. Each category corresponds with the score of each video from 1 to 5. These categories will be called labels hereafter. In order to implement the recognition system a learning process on the data described above must be previously applied. In order to perform a comparison between different classification techniques, three methods are under study: neural networks [42], decision trees [43] and Ameva algorithm [34].

The remainder of this section contains the methodology for the execution of each classification algorithms presented. First, neural network approach will be explained, where will be shown the structure during the training stage. Later, decision tree technique is presented, introducing the configuration of the tree and the features taken into account. Finally, a new approach based on Ameva discretization algorithm is presented. The latter algorithm is a new contribution to the field of electroencephalography signal analysis, due to signal will be processed in a discrete way, instead of the common continuous algorithms used in this area.

**Fig. 6:**
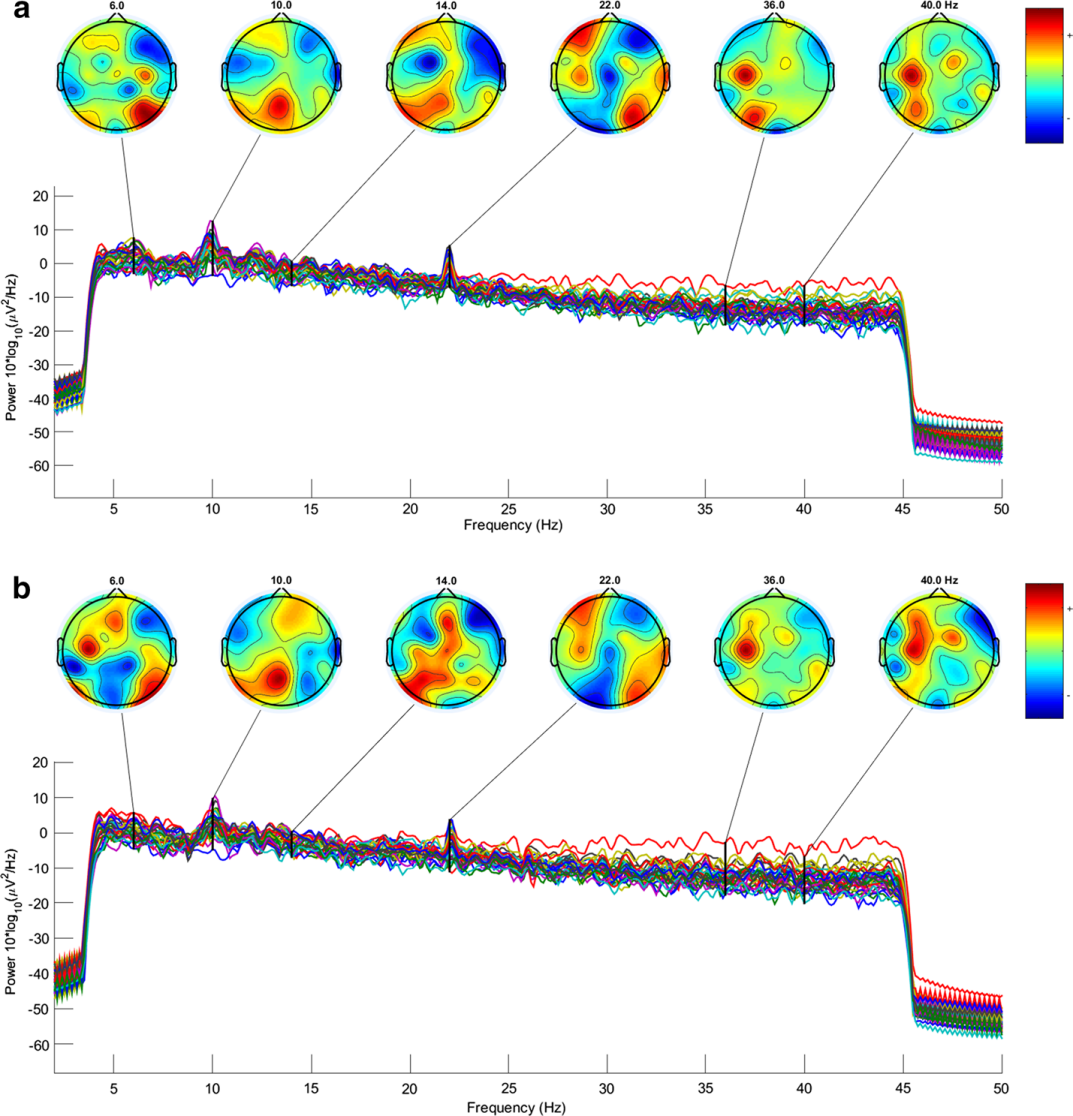
*PSD* mean values and scalp map obtained from neurological activity reactions during the visualization of videos with high and low scores. **a** PSD mean values and scalp map images associated to videos with rating higher than 3 (like), **b** PSD mean values and scalp map images associated to videos with rating lower than 3 (dislike)

**Fig. 7:**
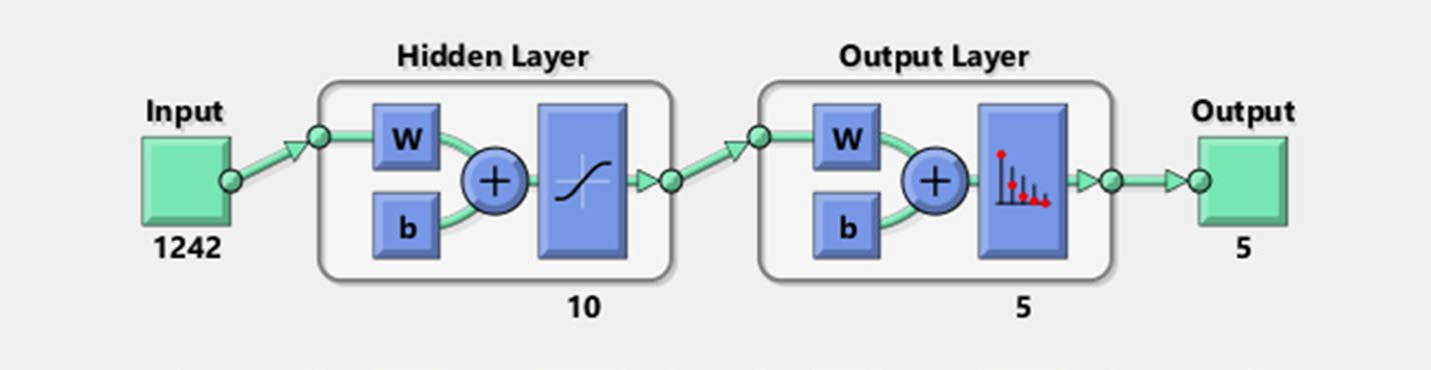
Neural network configuration with 10 neurons in the unique hidden layer

Although in this work statistics will be used as inputs for the classification system (also called features), Fig. 6 shows the scalp maps and *PSD* values for two groups. The first group, represented in Fig. 6, was obtained from ads with a rating higher than 3. Pay attention in the high brain activity for the sensor FC5 for low alpha band (6–8 Hz). However, the activation level of the same sensor in Fig. 6, which was obtained from ads with rating levels lower than 3, is lower for that band. Same happens with the O1 sensor. In this case, the neural activity in this area for ratings higher than 3, is significantly lower compared to low-rated ads. These effects will be detected by the learning algorithms applied in this section and, by hence, like/dislike rate recognition will be done using classical algorithms and discrete techniques.

### Neural network based approach

**Fig. 8:**
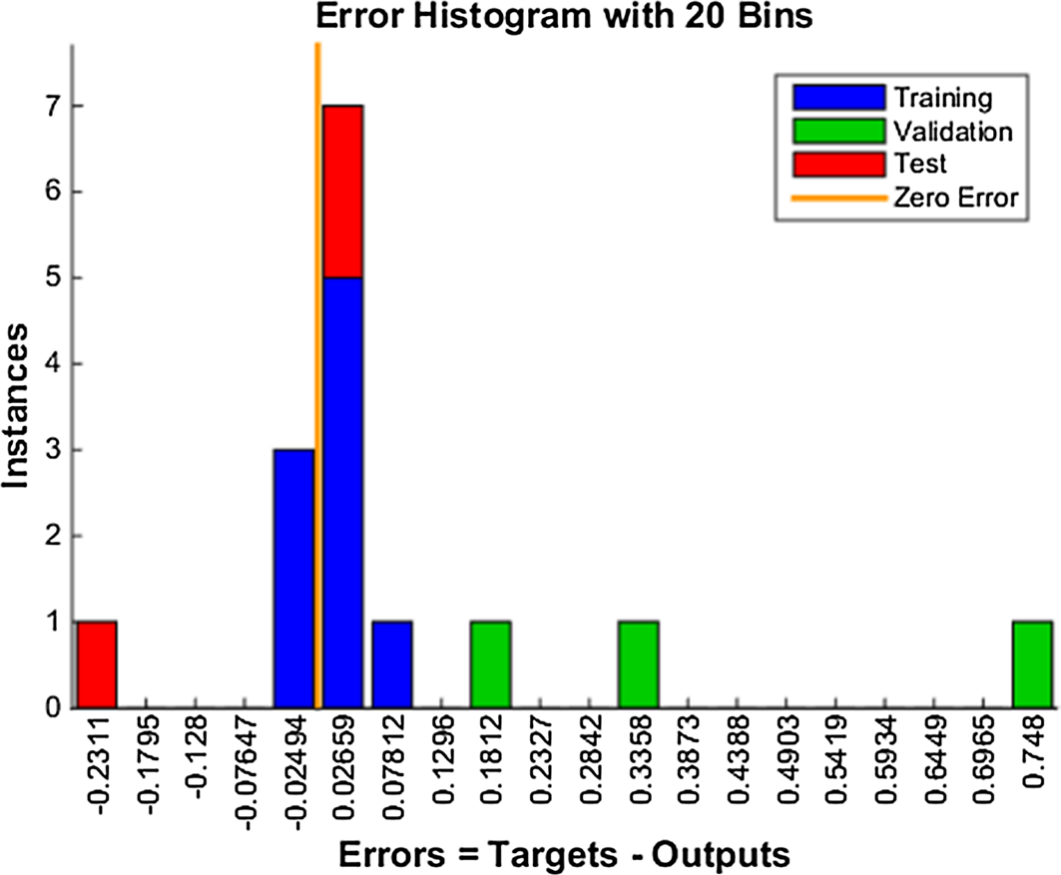
Neural network training error histogram

**Fig. 9:**
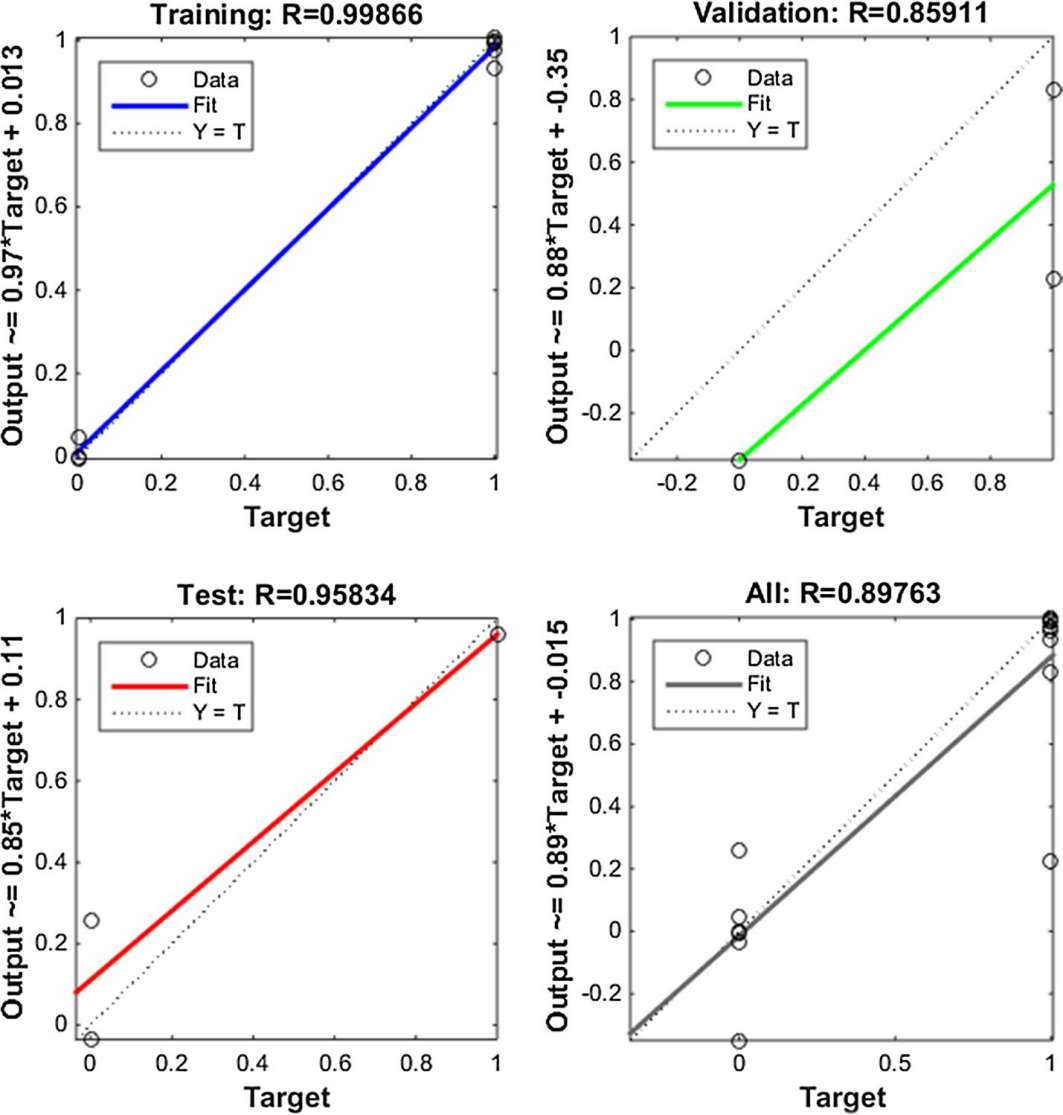
Linear regression of targets relative to outputs

In the first case, a neural network was built in order to classify the statistical inputs obtained from biometric parameters of the EEG described above. The network configuration, as shown in Fig. 7, is composed of two layers (one hidden and one output). The hidden layer consists on 10 neurons, whereas the output layer has only 5 neuron. Each neuron in the output layer is associated to a concrete class value. The class represented by the most active neuron will be the most likely score associated to that advertisement. This configuration has been obtained from set of 14 experiments and their results are shown in Fig. 8. Sigmoid neurons have been employed in both layers, the hidden and the output layers. The algorithm used for the training process was the Scaled Conjugate Gradient and the performance has been optimized by the Cross-Entropy function. Figure 9 shows the regression plot during the learning process.

### Decision tree based approach

**Fig. 10:**
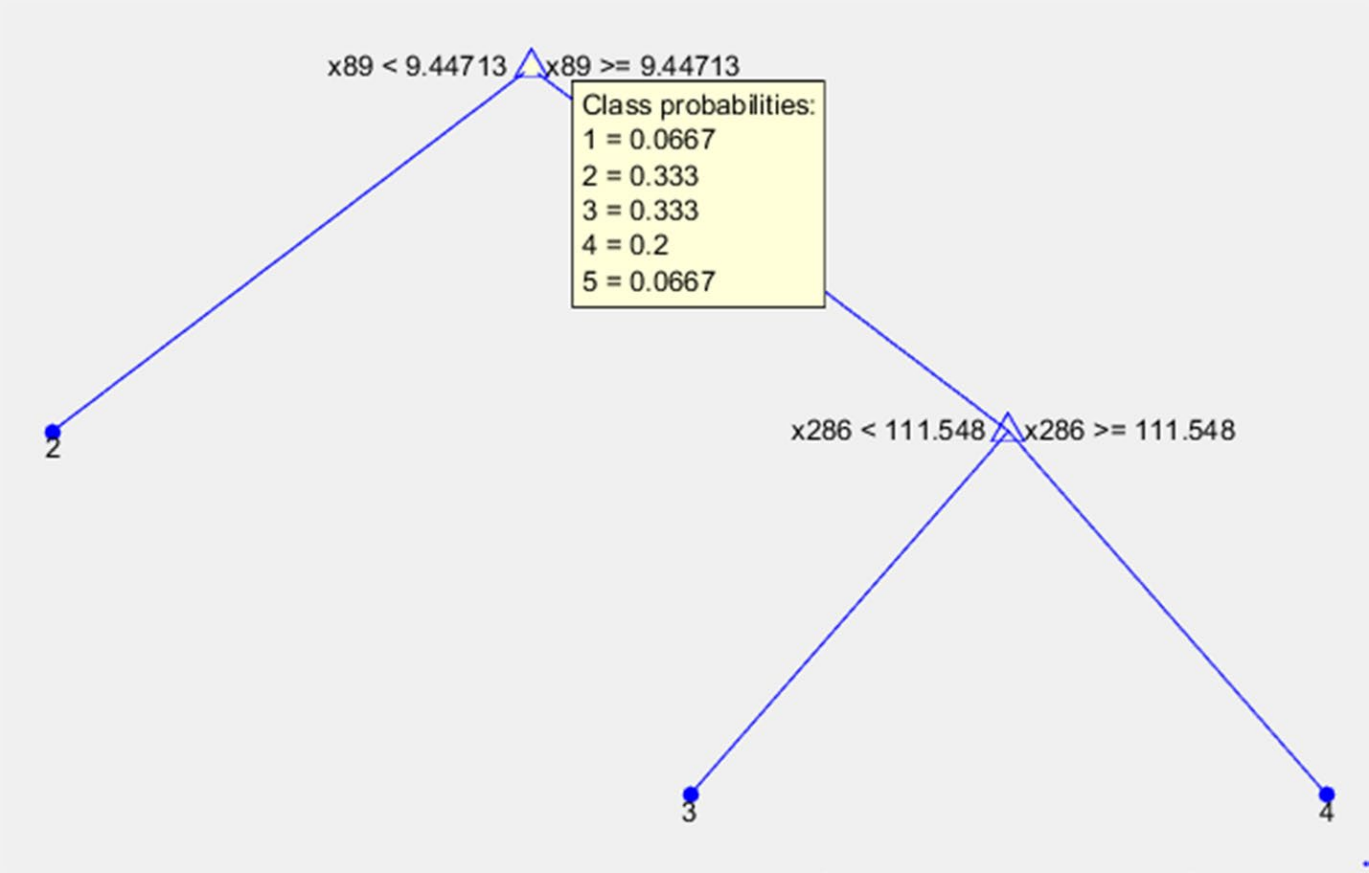
Binary tree topology once executed the C4.5 algorithm

On the other hand, in order to compare the new approach presented below, decision tree algorithm has been chosen due to it has been largely used in the literature. In this case, the classification algorithm C4.5 [25] was trained from the values and classes collected in the same dataset built previously. The topology of the tree generated by this algorithm is shown in Fig. 10. The average accuracy of this method is 71 %. Testing and cross-validation process used in this approach are the same previously used in ANN (Artificial Neural Networks) [23], so the results can be compared.

### Ameva based approach

**Fig. 11:**
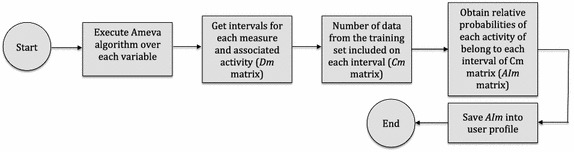
Overall process of data analysis and interval determination

Finally, a new approach based on discretization algorithm is presented in this section. In this case, the input values will be classified throughout continuous techniques. The new algorithm, based on Ameva, will be able to generate belonging intervals in which inputs will be clustered. Ameva algorithm can be defined as a binning method [44] which formal definition will be described below. Although binning techniques are mostly used to reduce the effects of minor observation errors by grouping a number of continuous values into a smaller number of intervals [45], Ameva method will be used to classify and discretize this information. Based on this groups or clusters, a fast and simple classification technique will be developed. In order to generate these intervals, inputs are splitted into time windows of fixed duration used to get training data and later, to obtain inputs for the recognition process. Each time window is composed by a set of EEG based on the inputs which were previously introduced. A performance analysis has been carried out in order to determine the optimum length of these windows. The maximum performance was obtained for 10 s time windows. Figure 11 shows the overall process of classification for the Ameva-based approach.

The first step of this process is the discretization of inputs (statistics) to reduce the computational cost of this algorithm. This discretization is based on Ameva algorithm [34]. This process relies on the maximization of the dependency relationship between the class labels $\mathcal { C }$ and the continuous-values attribute $\mathcal { L }( k )$, and at the same time minimizing the number of discrete intervals *k*.

**Fig. 12:**
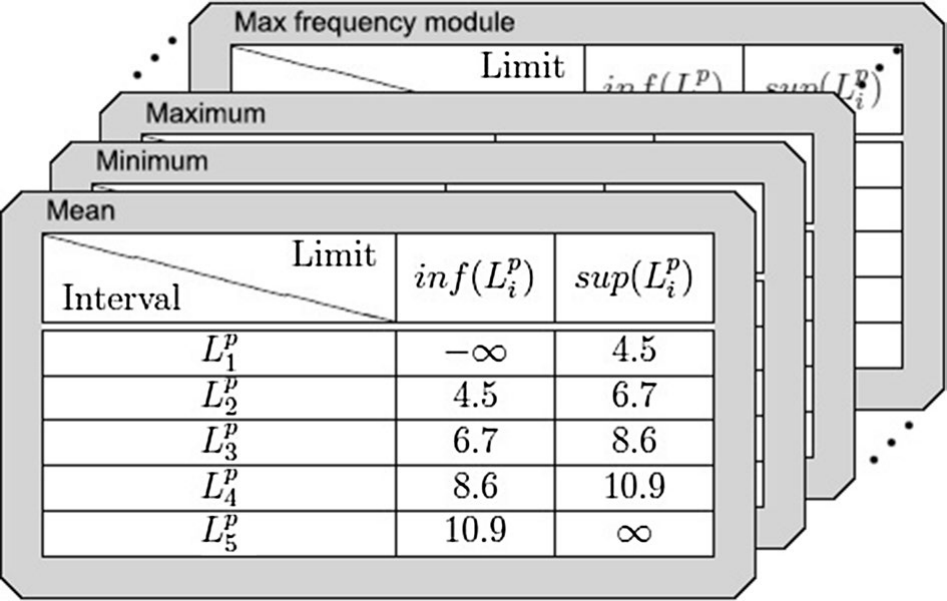
Discretization matrices

The application of the algorithm to each statistic of the system (arithmetic mean, minimum, maximum, etc.) allows obtaining a set of intervals associated with a particular $\mathcal { C }$ tag. Thus, after processing all system statistics, a matrix denoted by $Dm\{ \mathcal { C }, L^{ l, s }, S \}$ is produced as output (see Fig. 12).

The dimensions of the matrix are in the order: (i) the label $\mathcal { C }$ of the preference [1–5]; (ii) the interval $L_{ i } = ( L^{ l }_{ i }, L^{ u }_{ i } ]$ defining lower $L^{ l }_{ i }$ and upper limit $L^{ u }_ { i }$ of the range; (iii) the statistics measurement of the data (arithmetic mean, minimum, maximum, etc.).

After the data collection $\mathcal { X }$, the probability associated with the statistical data for each rating is computed. In order to carry out this process, a matrix denoted by $Cm\{ x, L_{ i}, S \}$ is defined as follows:

$$\begin{aligned} Cm_{ x, i, s } = | x \in \mathcal { X }|,\quad x \ge L^{ l }_{ i } \wedge x < L^{ u }_{ i } \wedge x{ \mathcal { C } } = C_{ s } \end{aligned}$$

It means that each entry of the matrix *Cm* contains the number of instances $x \in \mathcal { X }$ belongs to a specific interval of the range of a statistical *S*.

After *Cm* is computed, the relative probability matrix is carried out. This matrix is denoted by $AIm\{ x, L_{ i }, \mathcal { S } \}$ and it determines the likelihood that a given value *x* associated to an *S* statistic corresponds to a specific $C_{ i }$ score (label). This ratio is based on the quality of the discretization performed by Ameva. The contents of the array *AIm* is defined as follows

$$\begin{aligned} AIm_{ c, i, s } = \frac{ Cm_{ c, i, s } }{ total_{ c, s } } \cdot \frac{ 1 }{ l - 1 } \sum ^{ l }_{ j = 1, j \ne c } \left( 1 - \frac{ Cm_{ j, i, s } }{ total_{ j, s } } \right)\end{aligned}$$

where $total_{ c, s }$ is the total number of time windows of the training process labeled with the *c* score for the *S* statistic.

**Fig. 13:**
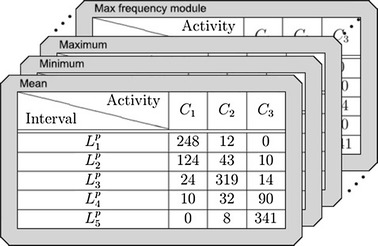
Class matrices

Figure 13 shows the contents of a real Class Matrix obtained during a learning process from all statistics. Within these 3 labels (reduced from original 5 to illustrate this example), five intervals determined by the Ameva algorithm can be observed in the Mean Class Matrix.

Finally, the overall process described on this section from matrix AIm is based on a majority voting system.

This process starts from the matrix *AIm* and uses a set of data $x \in \mathcal { X }$ for each of the statistics belonging to the set $\mathcal { S }$. The process consists of finding a rating $mpa \in \mathcal { C }$ such that the likelihood is maximized. The above rule is included in the expression

2 $$\begin{aligned} mpa( \mathcal { X } ) = \max \sum ^{ s }_{ s = 1 } AIm_{ c, i, s } | x_{ s } \in ( L^{ l }_{ i }, L^{ u }_{ i } ] \end{aligned}$$

The expression shows that the weight of each statistical metric to the calculation of the probability is equal. This can be carried out under the assumption that all statistics provide the same amount of information to the system, and that there is no correlation between them. Thus, the *mpa* represents those activities whose data is more fitted to the *AIm* set values using a majority voting system.

**Fig. 14:**
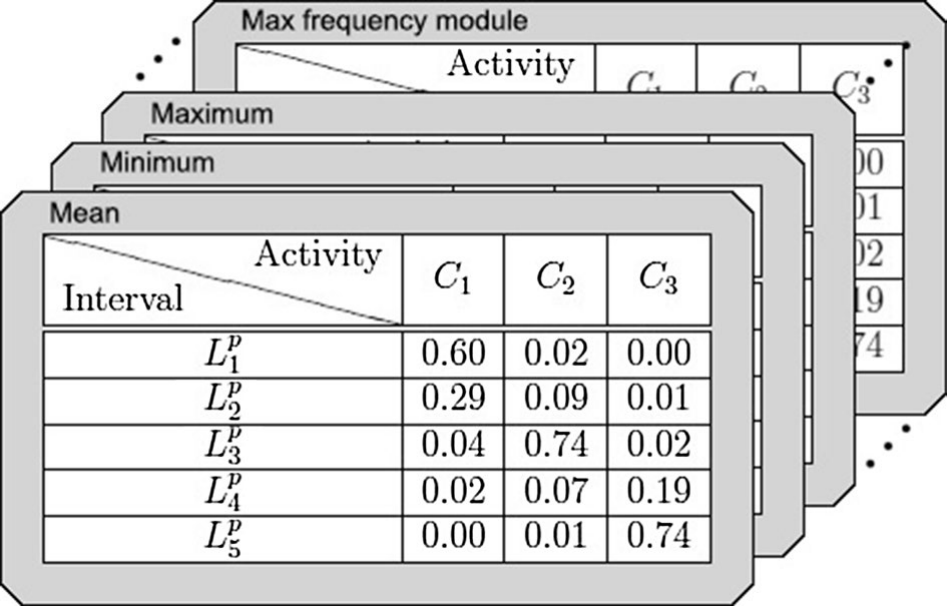
Liking-score-interval matrices

Figure 14 shows a set of values for each of the positions of the Liking-Score-Interval matrix. These results have been obtained from the training set of the Class Matrix described above in Fig. 13.

The final system with an innovative algorithm has been developed and deployed in a smartphone to get the necessary data to identify the rating associated with the video that the user is watching. Although it could be though that it does not make sense to integrate this development into a smartphone, it should be taken into account that next generation of EEG devices (Emotiv Insight) or even devices currently on sale (MindWave), can be paired with the smartphone and they are able to share real time information between the EEG device and the smartphone. In this work, Emotiv EPOC was connected to an Android device [46] using the SBS2, which is freely available under the MIT License on GitHub at https://github.com/SmartphoneBrainScanner. However, although it is possible to connect the Emotiv EPOC to the smartphone, no Android API is provided by Emotiv Systems to work with. In the next section, an optimization of the original Ameva algorithm is presented and a comparative between two versions will be done.

## Description of the Ameva system

The main advantage of this approach is the reduction of the battery consumption derived from the usage of discrete variables instead of continuous inputs. Furthermore, the dependencies between them are removed from the system to get just the information and reducing the noise.

In order to improve this efficiency and reduce even more the battery consumption caused by classification algorithms, several improvements over the original algorithm has been applied. The discretization process performed in the original algorithm needs to evaluate the cuts criterion using an iterative algorithm. By hence, its goal was to find the cut that minimizes the variance of the class labels belonging to the instances of each interval. The application of the algorithm to a multivariate dataset with a high amount of samples lacks low performances and a high complexity.

The optimization introduced allows to simplify and reduce the complexity. The new algorithm computes the sample variance of the class label associated with each instance only once. This optimization generates more intervals than the original approach. However, the intervals generated by the original algorithm are a subset of those generated by the optimization, which ensures that the results for the classification process are theoretically a superset of the intervals produced by the original algorithm. In practice, the number of intervals resulting from both approaches are really close each other. In terms of runtime, a significant reduction was expected by the application of the improvement described. These expectations were met in the testing process, where execution time was reduced by 50 %. Table 2 shows the comparison between both algorithm (Ameva original and Ameva optimized) in terms of runtime from a dataset of 1280 instances and 1242 statistics.

**Table 2:** Comparison between Ameva original and Ameva optimized algorithms

# of features	Ameva original	Ameva optimized	Ameva original	Ameva optimized
	Time (s)	Time (s)	# of intervals	# of intervals
1	0.48	0.12	9	17
5	1.67	0.64	48	86
10	2.92	1.43	78	121
20	4.87	2.29	120	215
50	13.57	6.78	317	634
100	25.16	11.97	624	1464
200	49.56	24.45	1214	4263
400	79.59	40.32	2312	7423
800	154.84	75.32	4788	14,263
1200	296.41	159.16	9421	30,125

## Results

For the validation stage, all classification algorithms were trained and tested under the same conditions, with identical configurations for the cross validation folds. For this purpose, a set of 10-folds cross validation with 70 % of training data, 15 % of validation and 15 % of testing has been generated. Tables 3 and 4 show the results of the comparison using the three methods presented above. Rows show the different classes based on the rating of each advertisement [1–5]. By the other side, columns present the details associated with each classification methods under testing (ANN, C4.5 and Ameva). First column shows the number of advertisement well-classified for each class using ANN. Second column presents the number of advertisements misclassified by this algorithm. Same parameters are collected from C4.5 algorithm, which are shown in the third and fourth column. Two more columns (fifth and sixth) present the performance analysis for Ameva method. Finally, the last column gathers the count of instances in the corresponding class.

**Table 3:** Classification results for short advertising movies from EEG inputs

Class	ANN success	ANN errors	C4.5 success	C4.5 errors	Ameva success	Ameva errors	Total instances
1	75	15	65	25	70	20	90
2	90	20	80	30	85	25	110
3	60	20	55	25	55	25	80
4	135	30	110	55	130	35	165
5	95	25	80	40	85	35	120
Summary	455	110	390	175	425	140	565

**Table 4:** Classification results for short advertising movies from EEG inputs

Class	ANN accuracy (%)	C4.5 accuracy (%)	Ameva accuracy (%)
1	83	72	77
2	81	72	78
3	75	68	68
4	81	66	78
5	79	66	70
Summary	80	69	75

**Table 5:** Analysis of the statistical significance of the differences in the accuracy averages among ANN, C4.5 and Ameva methods considering a significance level of 5 %

Class	ANN/C4.5		ANN/Ameva		C4.5/Ameva	
1	1.793	3.65 %	0.942	17.32 %	0.861	19.47 %
2	1.609	5.38 %	0.836	20.17 %	0.778	21.81 %
3	0.879	18.97 %	0.879	18.97 %	0.000	50.00 %
4	3.147	0.08 %	0.692	24.44 %	2.472	0.67 %
5	2.179	1.47 %	1.491	6.80 %	0.696	24.31 %
All classes	4.452	0.00 %	2.150	1.58 %	2.322	1.01 %

**Table 6:** Comparison of execution time between Ameva optimized, ANN and C4.5 algorithms from a dataset with an incremental size of inputs and 1280 instances

# of features	Ameva optimized	Artificial neural networks	Decision tree (C4.5)
	Time (s)	Time (s)	Time (s)
1	0.12	0.34	0.21
5	0.64	1.23	0.89
10	1.43	2.13	1.89
20	2.29	4.75	3.15
50	4.78	9.81	8.41
100	9.97	17.83	13.47
200	18.45	34.51	38.63
400	37.32	60.18	43.73
800	75.32	119.51	83.13
1200	129.16	235.65	158.32

As can be seen in Table 4, the accuracy reached by the method of classification based on Ameva algorithm is 75 % for all rating. Compared to other classification algorithms such as ANN or C4.5, Ameva technique is located above C4.5, increasing accuracy by 6 % over that, and below ANN with a difference of 5 %. An analysis of the statistical significance of accuracy among the three procedures is given in the Table 5. In this table can be seen considering a significance level of 5 %, that:

- About the ANN and C4.5 approaches comparison, there are significant differences for the classes 1, 4, and 5, and when all classes are considering.
- With respect to the ANN and Ameva approaches comparison, there are not significant difference for each class. Nevertheless, if all classes are considered, clear differences in the accuracy can be observed.
- Regarding the Ameva and C4.5 approaches comparison, there are significant differences for the class 4, and when all classes are considered.

On the one hand, both learning and classification are significantly faster than other established algorithms compared in this paper. This means that in both energy consumption and overloading, the proposed system is placed in a better position than other algorithms. This improvement in training time can be seen in Table 6. On the other hand, the results obtained from Ameva-based technique are more clearly than results obtained in ANN, among other learning algorithms. This is because of the results of Ameva-based approach are simple intervals and no dimensionality changes are needed. Because the algorithm C4.5 is hierarchical, it is not possible to exploit the potential of parallelism when evaluating different conditions. That is, due to there are different paths from the root to the leaves, and being conditions dependent of these paths, the path followed to reach the leaves (labels) must be linear (regardless of the complexity of the classification system, that is logarithmic). However, in the case of Ameva, membership of inputs to intervals generated during the learning process is completely independent of the feature selected. Thus, all conditions can be evaluated in parallel to obtain the coefficient of belonging to the label. This, and considering that the recognition process was executed on mobile devices with four or more cores, makes the chances of parallelism completely exploitable. This makes that the recognition system can be ran into a smartphone or any other wearable and the time won’t be a problem.

**Fig. 15:**
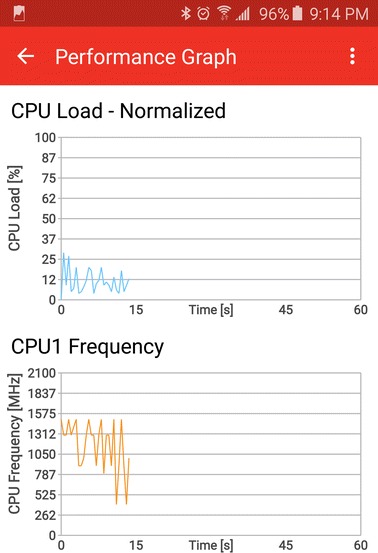
Trepn profiling tool screenshot

The learning phase of the process has been developed using MATLAB R2014b. The classification process of different approaches has been implemented on different devices by using Android Studio and the SDK tools provided by Google. In order to obtain the energy consumption comparison, the monitoring software Trepn Profiler has been used. Trepn Profiler is an on-target power and performance profiling application for mobile devices distributed by Qualcomm for monitoring snapdragon processors. This diagnostic tool allows profiling of the performance and power consumption of Android applications running under this family of processors. Figure 15 shows a screenshot of this tool. As can be observed in this figure, different CPU frequencies are being monitored. The test process was conducted on Google Nexus S, Samsung Galaxy S4, LG Nexus 5 and Google Nexus One devices.

## Conclusions

In this paper a system of recognition of likes/dislikes for TV short video advertisements using electroencephalographic data has been presented. The system proposed can automatically determine, through prior learning, whether users like the advertisements displayed in real time and reducing the time consumption in more than 20 % compared to other classical algorithms such as C4.5 and ANN. Under certain circumstances, the improvement of the algorithm presented in this paper is up to 40 %. These benefits have been obtained during the classification process, once the system has been trained using the corresponding algorithm. This new approach based on Ameva algorithm generates a set of intervals for each statistic, which allows to discretize the inputs of the system. From these intervals, a simple and fast classification algorithm has been presented. In order to evaluate the solution and determine the best set of input parameters, a dataset consisting of 1242 statistics from 14 EEG channels has been generated using the Emotiv EPOC device. Three different algorithms, artificial neural network (ANN), classification tree and Ameva based method, have been used. In the first case, an accuracy of approximately 80 % was obtained, while in the second case the accuracy is lowered to 69 % on average. Finally, Ameva method present results closer to the ANN method (75 %), but using a discrete approach instead. This approach have a positive impact in time consumption and complexity of the solution. Although the results in terms of accuracy are not higher than those obtained by ANN approach, this methodology provides a substantial increment of the battery life, decreasing the recharges needed during a day for pervasive systems using this technology. It should be taken into account the typology of the EEG headset used in this work, Emotiv EPOC, that represents an important step towards the inclusion of these systems in the users’ daily life. It is a low-cost ($ 400 approximately) that can be used without any restrictions and in a comfortable way, unlike the headsets used in other related works with higher prices (more that $ 10,000) and higher spacial resolutions.
